# Supplementary material for: Dendrite injury triggers neuroprotection in Drosophila models of neurodegenerative disease
Source: Sci Rep. 2024 Oct 21;14:24766. doi: 10.1038/s41598-024-74670-4 (PMC11494097; doi:10.1038/s41598-024-74670-4)
Supplement: Supplementary file 1 — Supplementary Information. [file 41598_2024_74670_MOESM1_ESM.pdf]

Dendrite injury, but not axon injury, triggers neuroprotection in Drosophila models of neurodegenerative disease.

Sydney E. Prange[1,3], Isha N. Bhakta[1], Daria Sysoeva[1], Grace E. Jean[1], Anjali Madisetti[1], Hieu H. N. Le[1], Ly U. Duong[1], Patrick T. Hwu[1], Jaela G. Melton[2], Katherine L. Thompson-Peer[1,2,3,4,5]*.

[1]Department of Developmental and Cell Biology, University of California Irvine, CA, USA

[2]Center for the Neurobiology of Learning and Memory, Irvine, CA

[3]Sue and Bill Gross Stem Cell Research Center, Irvine, CA

[4]Reeve-Irvine Research Center, Irvine, CA

[5]Lead Contact

*Correspondence: ktpeer@uci.edu

Supplementary Figures, Supplementary Figure legends, and Supplementary Methods

Supplemental Figure 1. Class IV da neurons overexpressing pathogenic polyglutamine transgenes experience progressive dendrite degeneration

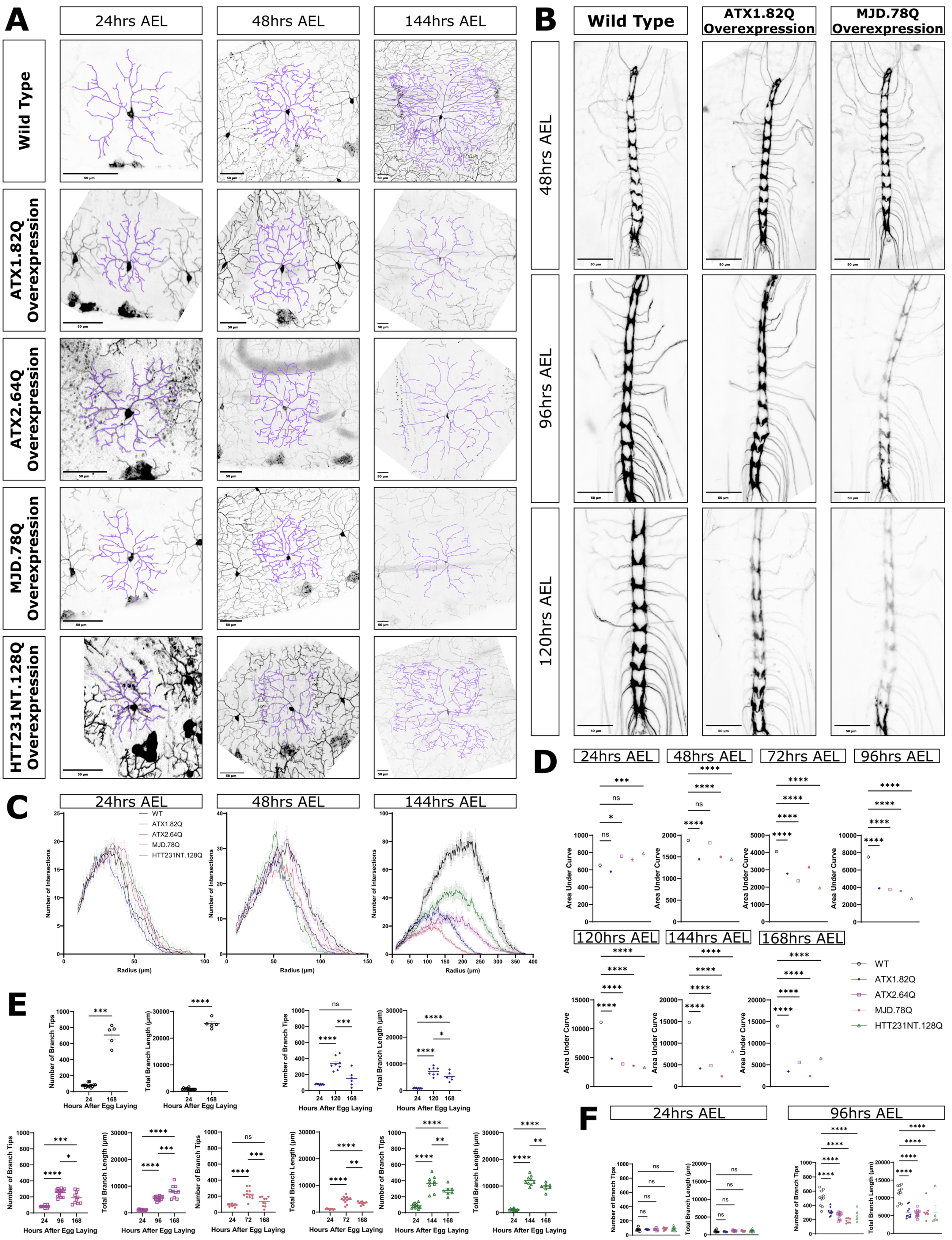

Supplemental Figure 1. Class IV da neurons overexpressing pathogenic polyglutamine transgenes experience progressive dendrite degeneration A) WT neurons and neurons overexpressing ATX1.82Q, ATX2.64Q, MJD.78Q, and HTT231NT.128Q at 24, 48, and 144 hrs AEL. Scale bar 50 µm. B) Ventral nerve cord for WT neurons, and neurons overexpressing ATX1.82Q and MJD.78Q at 48, 96, and 120 hrs AEL. Scale bar 50 µm. C) Sholl analysis at 24, 48, and 144 hrs AEL for WT neurons, and neurons overexpressing ATX1.82Q, ATX2.64Q, MJD.78Q, and HTT231NT.128Q. Legend applies for all graphs in C. D) Area under the Sholl curve at 24-168 hrs AEL for WT neurons and neurons overexpressing ATX1.82Q, ATX2.64Q, MJD.78Q, and HTT231NT.128Q. Legend applies for all graphs in D, E, and F. Mean ± SEM. One-way ANOVA with Dunnett's multiple comparisons correction. E) Starting and endpoint values for dendrite branches and length for WT neurons (top left). Welch's t-test. Starting, maximum, and endpoint values for dendrite branches and length for ATX1.82Q neurons (top right), ATX2.64Q neurons (bottom left), MJD.78Q neurons (bottom middle), and HTT231NT.128Q neurons (bottom right). One-way ANOVA with Tukey's multiple comparisons correction. F) Dendrite branches and length at 24 hrs AEL and 96 hrs AEL between WT neurons and ATX1.82Q, ATX2.64Q, MJD.78Q, and HTT231NT.128Q neurons. One-way ANOVA with Dunnett's multiple comparisons correction.

Supplemental Figure 2. Overexpression of pathogenic polyQ proteins does not cause degeneration in class I ddaE neurons, and only the most pathogenic polyQ proteins cause degeneration in class III ddaA neurons.

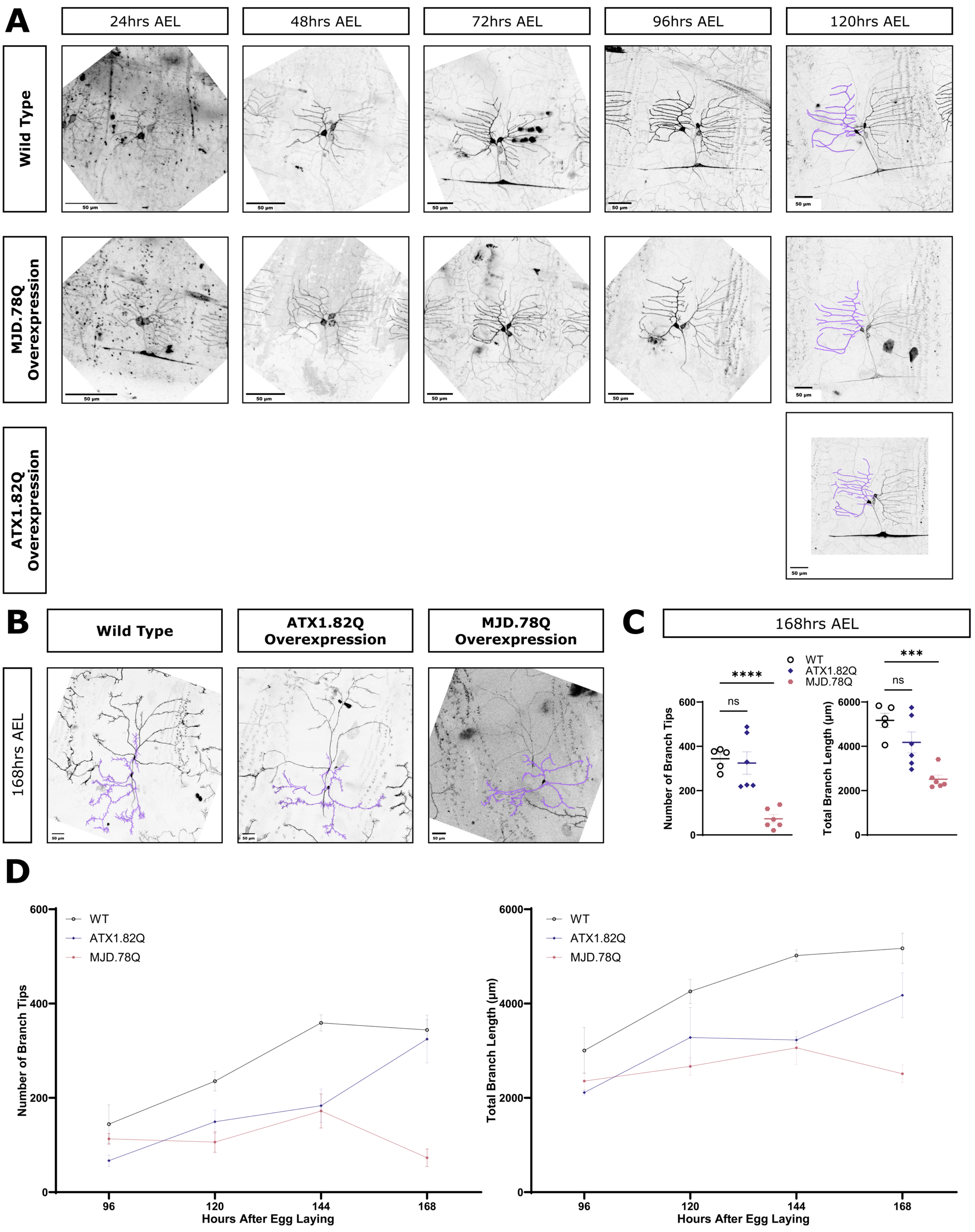

Supplemental Figure 2. Overexpression of pathogenic polyQ proteins does not cause degeneration in class I ddaE neurons, and only the most pathogenic polyQ proteins cause degeneration in class III ddaA neurons. A) Class I ddaE WT and MJD.78Q overexpression neurons at 24-120 hrs AEL and ATX1.82Q overexpression neurons at 120 hrs AEL. Scale bar 50 μm. B) Class III ddaA WT and ATX1.82Q and MJD.78Q overexpression neurons at 168 hrs AEL. Scale bar 50 μm. C) Number of branches and length at 168 hrs AEL for class III ddaA WT neurons and ATX1.82Q and MJD.78Q overexpression neurons. D) Number of branches and total branch length at 96-168 hrs AEL for class III ddaA WT neurons and ATX1.82Q and MJD.78Q overexpression neurons. Mean ± SEM.

Supplemental Figure 3. Pathogenic polyglutamine protein expression does not prevent dendrite regeneration.

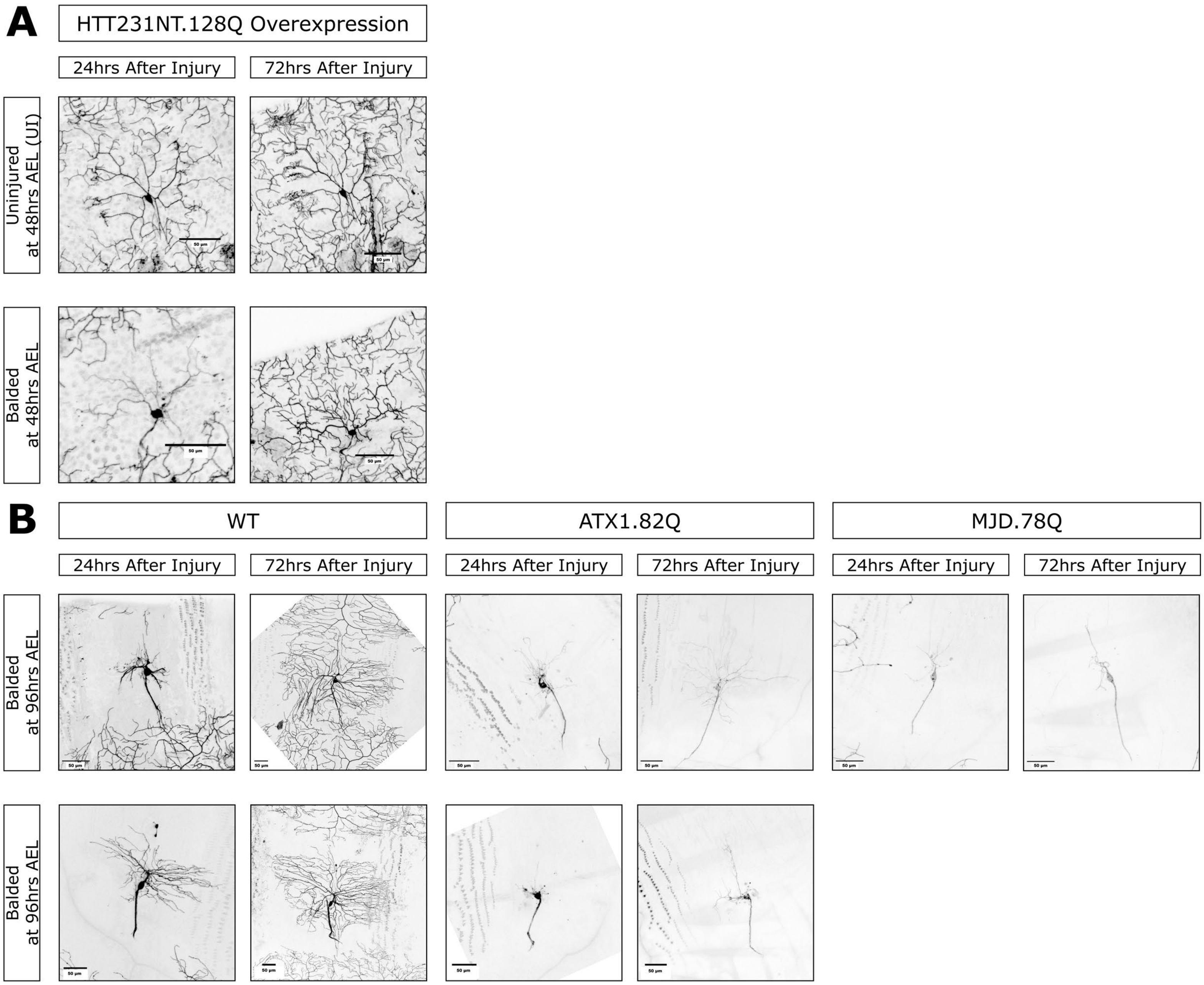

Supplemental Figure 3. Pathogenic polyglutamine protein expression does not prevent dendrite regeneration. A) Uninjured and balded at 48 hrs AEL HTT231NT.128Q overexpression neurons at 24 and 72 hrs after injury. Scale bar 50 µm. B) WT, ATX1.82Q overexpression, and MJD.78Q overexpression neurons balded at 96 hrs AEL at 24 and 72 hrs after injury. For WT and ATX1.82Q two representative images are shown at each time point representing results from two different animals. Scale bar 50 µm.

Supplemental Figure 4. Uninjured age-matched control neurons for single dendrite branch injury experiments.

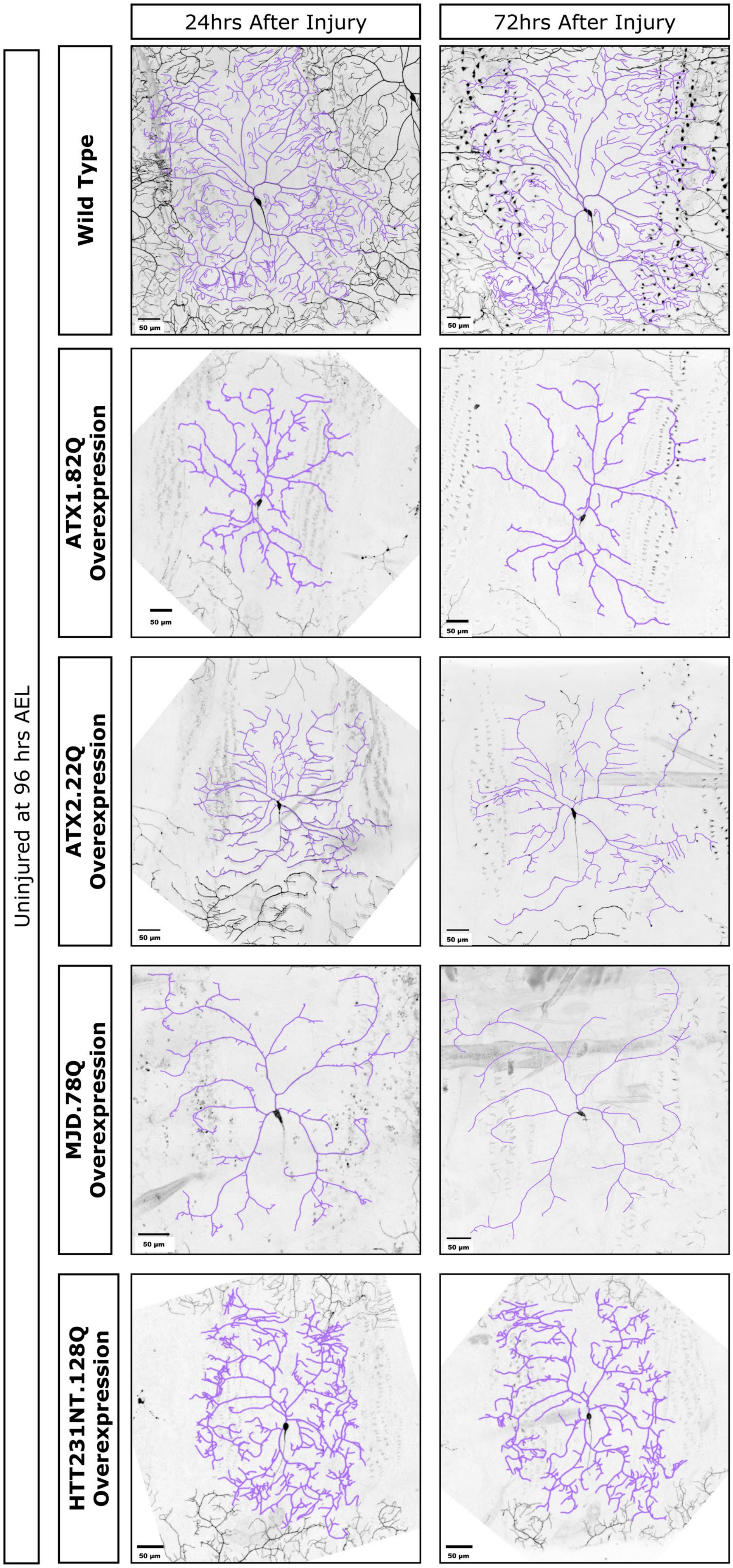

Supplemental Figure 4. Uninjured age-matched control neurons for class IV ddaC single dendrite branch injury experiments. Uninjured class IV ddaC neurons corresponding to neurons in Figure 3 at 24 and 72hrs after injury for WT and ATX1.82Q, ATX2.64Q, MJD.78Q, and HTT231NT.128Q overexpression neurons. Scale bar 50 µm.

Supplemental Figure 5. Location of single branch injury does not guide location of new growth.

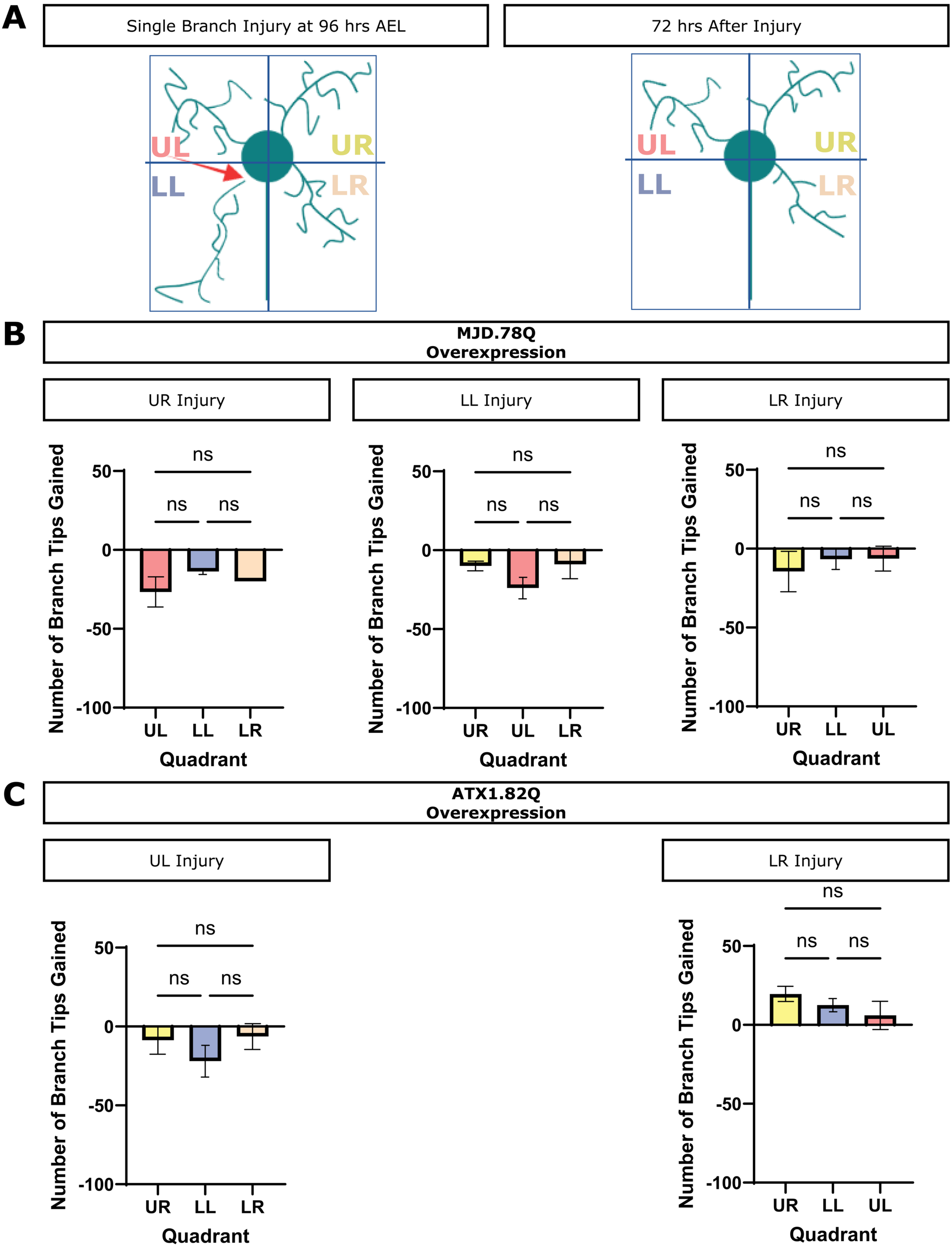

Supplemental Figure 5. Location of single branch injury does not guide location of new growth. A) Schematic demonstrating quadrants for analysis of arbor growth location following SBI in class IV ddaC neurons. UL = upper left, LL = lower left, UR = upper right, LR = lower right. Red arrows represent 2 photon laser injury. B) Number of branch tips gained for different quadrants of the dendrite arbor following SBI in another quadrant for MJD.78Q and ATX1.82Q overexpression class IV ddaC neurons. One-way ANOVA with Tukey's multiple comparisons correction.

Supplemental Figure 6. Non-pathogenic poly-Q neurons, like WT neurons, mildly respond to single branch injury.

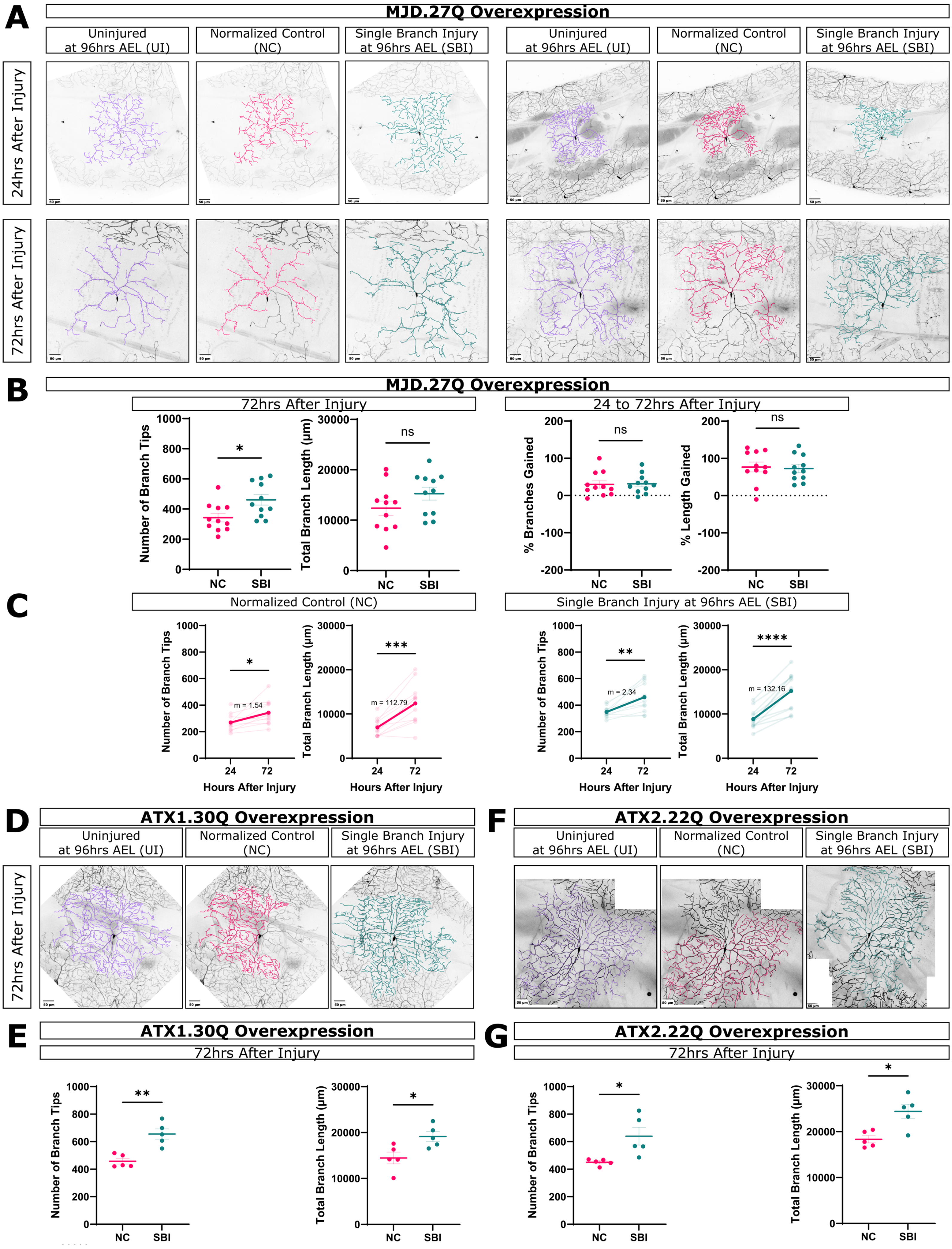

Supplemental Figure 6. Non-pathogenic poly-Q neurons, like WT neurons, mildly respond to single branch injury. A) Uninjured, normalized control, and single branch injured class IV ddaC MJD.27Q overexpression neurons at 24 and 72 hrs after injury. Two pairs of this data are shown representing two separate animals to show variation in the degenerative phenotype. Scale bar 50 μm. B) Comparing number of branch tips and total branch length of NC and SBI class IV ddaC MJD.27Q overexpression neurons at 72 hrs after injury or comparing % change in number of branch tips and total branch length between 24 to 72 hrs after injury between NC and SBI class IV ddaC MJD.27Q overexpression neurons. Welch's t-test. C) Number of branch tips and total branch length at 24 and 72 hrs after injury for NC and SBI class IV ddaC MJD.27Q overexpression neurons. Individual neurons are faded, solid lines represent the average slope (m) between the mean 24 and 72 hour after injury values. Paired t-test. D) Uninjured, normalized control, and single branch injured class IV ddaC ATX1.30Q overexpression at 72 hrs after injury. Scale bar 50 μm. E) Comparing number of branch tips and total branch length of NC and SBI class IV ddaC neurons at 72 hrs after injury between NC and SBI class IV ddaC ATX1.30Q overexpression neurons. Welch's t-test. F) Uninjured, normalized control, and single branch injured class IV ddaC ATX2.22Q overexpression at 72 hrs after injury. Scale bar 50 μm. G) Comparing number of branch tips and total branch length of NC and SBI class IV ddaC neurons at 72 hrs after injury between NC and SBI class IV ddaC ATX2.22Q overexpression neurons. Welch's t-test.

Supplemental Figure 7. Single branch injury induces neuroprotection in hMAPT overexpression neurons.

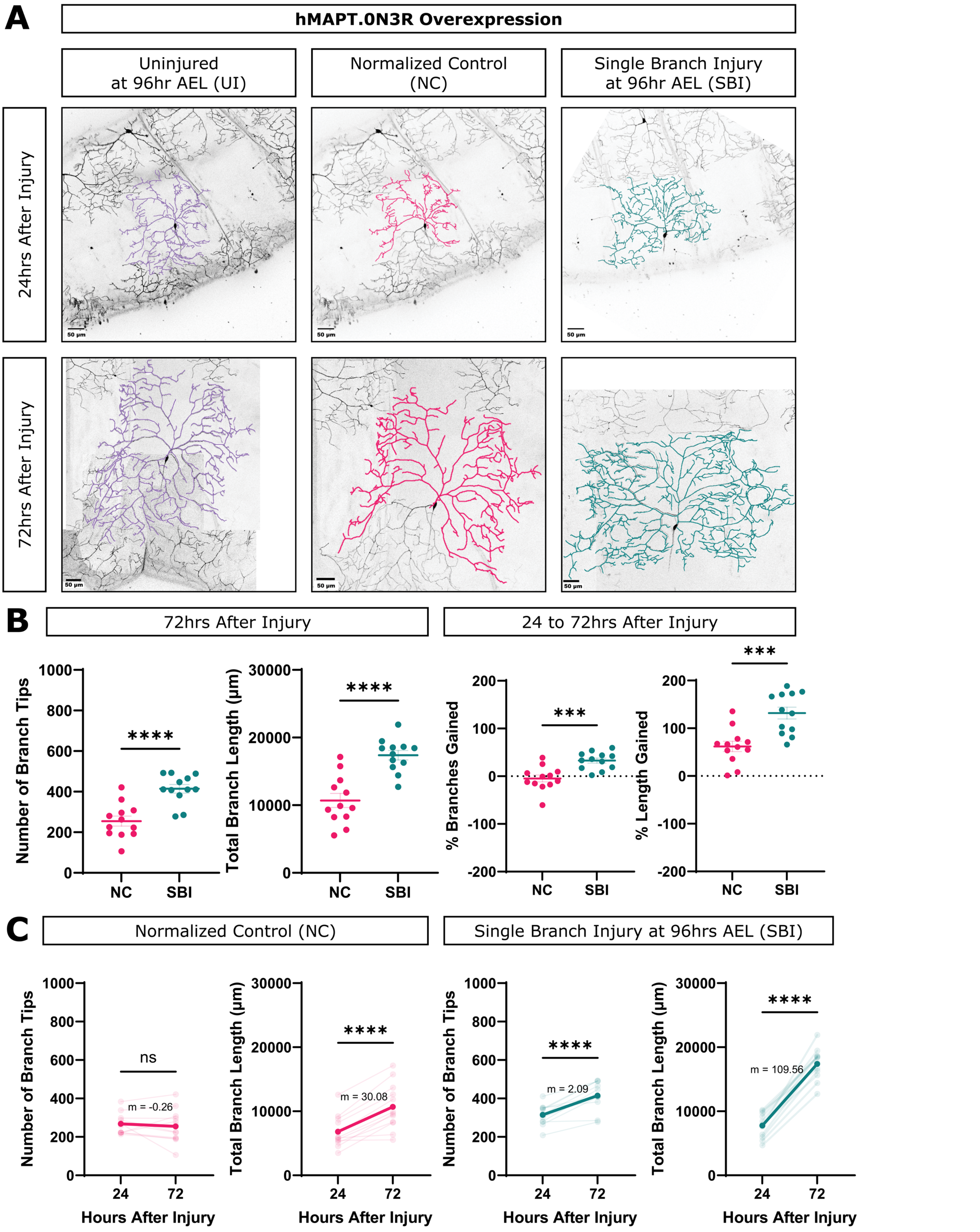

Supplemental Figure 7. Single branch injury induces neuroprotection in hMAPT overexpression neurons. A) Uninjured, normalized control, and single branch injured class IV ddaC hMAPT.0N3R overexpression neurons at 24 and 72 hrs after injury. B) Comparing number of branch tips and total branch length of NC and SBI class IV ddaC neurons at 72 hrs after injury or comparing % change in number of branch tips and total branch length between 24 to 72 hrs after injury between NC and SBI class IV ddaC hMAPT.0N3R overexpression neurons. Welch's t-test. C) Number of branch tips and total branch length at 24 and 72 hrs after injury for NC and SBI class IV ddaC hMAPT.0N3R overexpression neurons. Individual neurons are faded, solid lines represent the average slope (m) between the mean 24 and 72 hour after injury values.

Supplemental Figure 8. Single branch injury induces recovery of branch length but not branch number in class III ddaA MJD.78Q overexpression neurons.

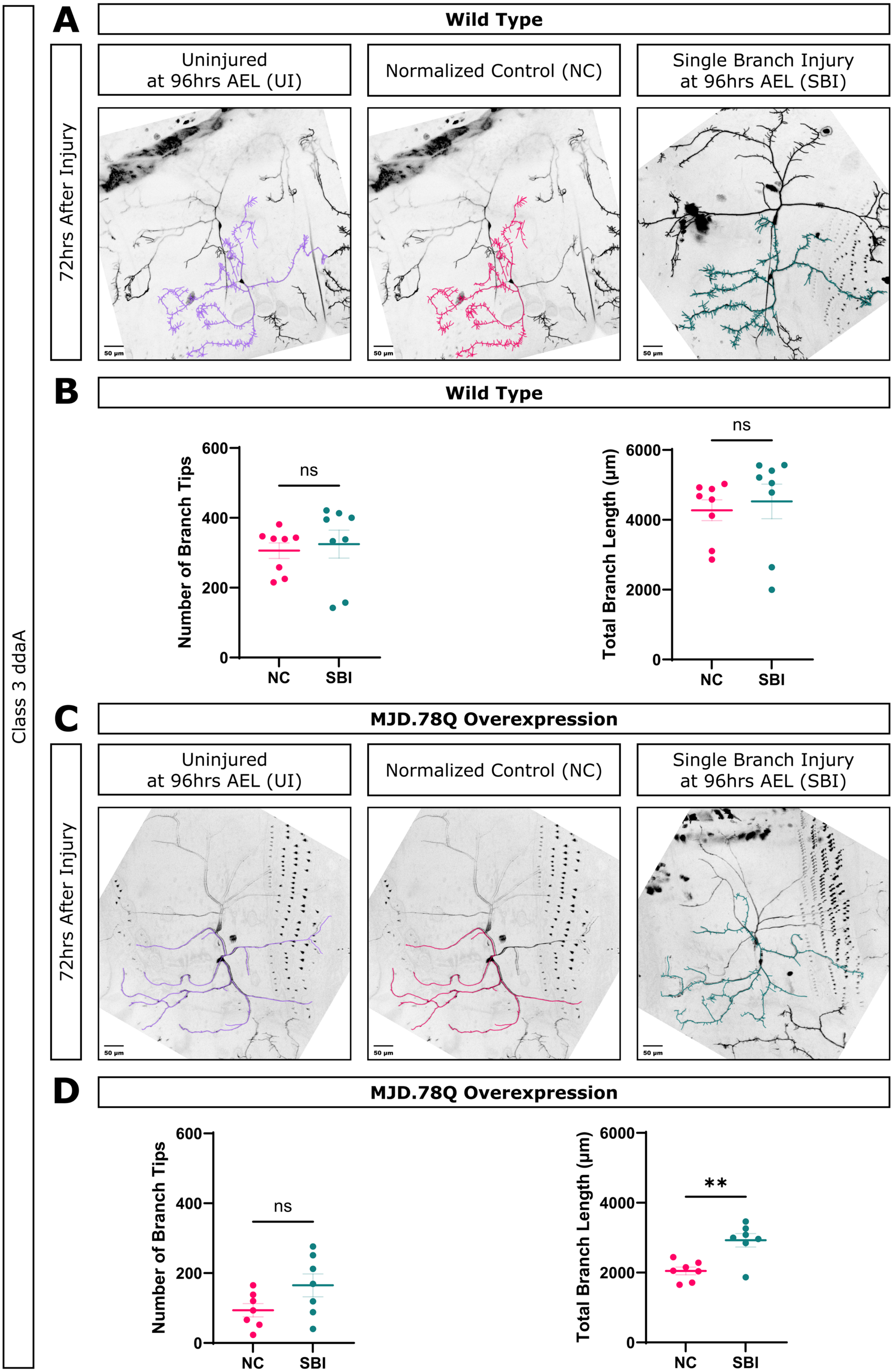

Supplemental Figure 8. Single branch injury induces recovery of branch length but not branch number in class III ddaA MJD.78Q overexpression neurons. A) Uninjured, normalized control, and single branch injured class III ddaA WT neurons at 72 hrs after injury. Scale bar 50 µm. B) Comparing number of branch tips and total branch length of class III ddaA NC and SBI WT neurons at 72 hrs after injury. Welch's t-test. C) Uninjured, normalized control, and single branch injured class III ddaA MJD.78Q overexpression neurons at 72 hrs after injury. Scale bar 50 µm. D) Comparing number of branch tips and total branch length of class III ddaA NC and SBI MJD.78Q overexpression neurons at 72 hrs after injury. Welch's t-test.

Supplemental Figure 9. Pathogenic polyQ model neurons degenerate dendrites after axon injury.

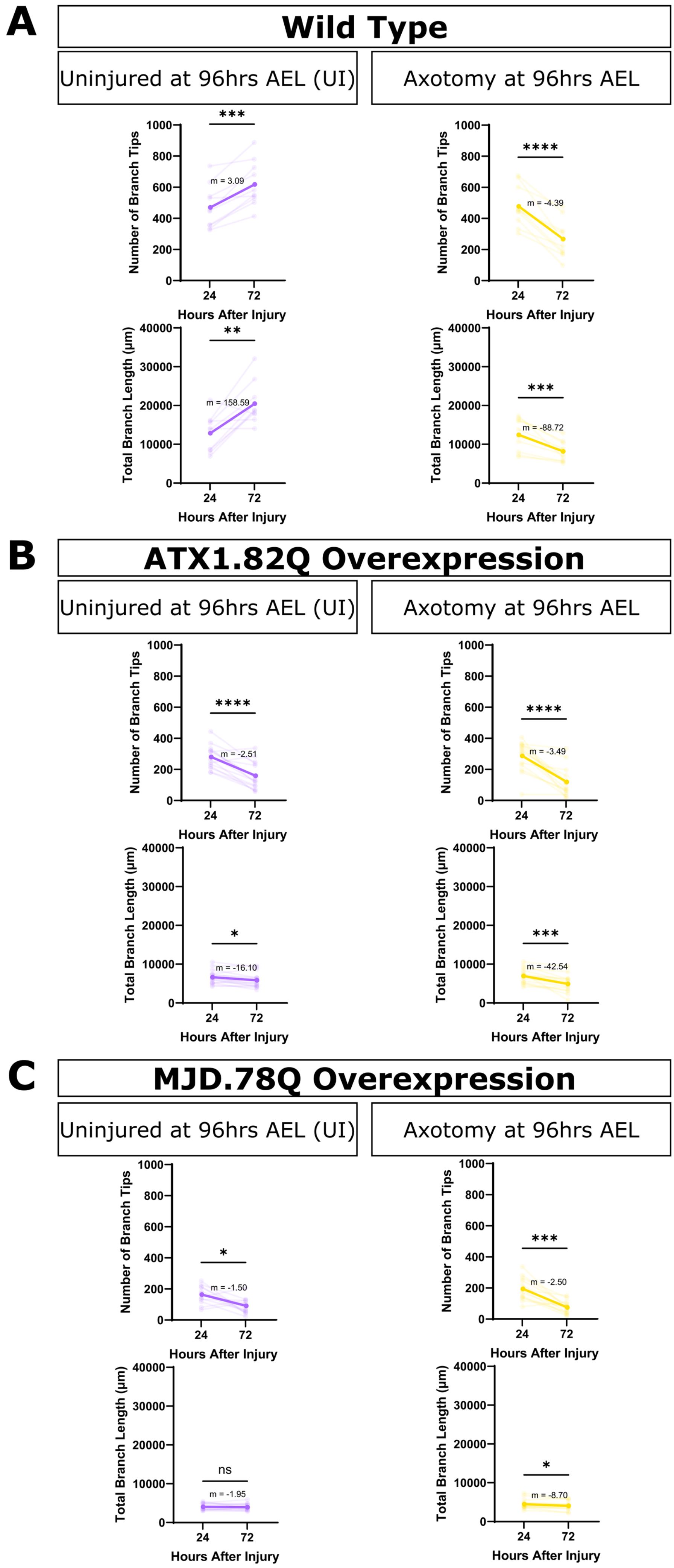

Supplemental Figure 9. Pathogenic polyQ model neurons degenerate dendrites after axon injury. A) Number of branch tips (top) and total branch length (bottom) at 24 and 72 hrs after injury for uninjured (purple) and axotomized (yellow) WT neurons. Paired t-test. B) Number of branch tips (top) and total branch length (bottom) at 24 and 72 hrs after injury for uninjured (purple) and axotomized (yellow) ATX1.82Q overexpression neurons. Paired t-test. C) Number of branch tips (top) and total branch length (bottom) at 24 and 72 hrs after injury for uninjured (purple) and axotomized (yellow) MJD.78Q overexpression neurons. Paired t-test.

Supplemental Figure 10. Neither microtubule composition nor dynamics are rescued in single branch injured pathogenic polyQ neurons and f-actin is not affected in single branch injured non-pathogenic polyQ neurons.

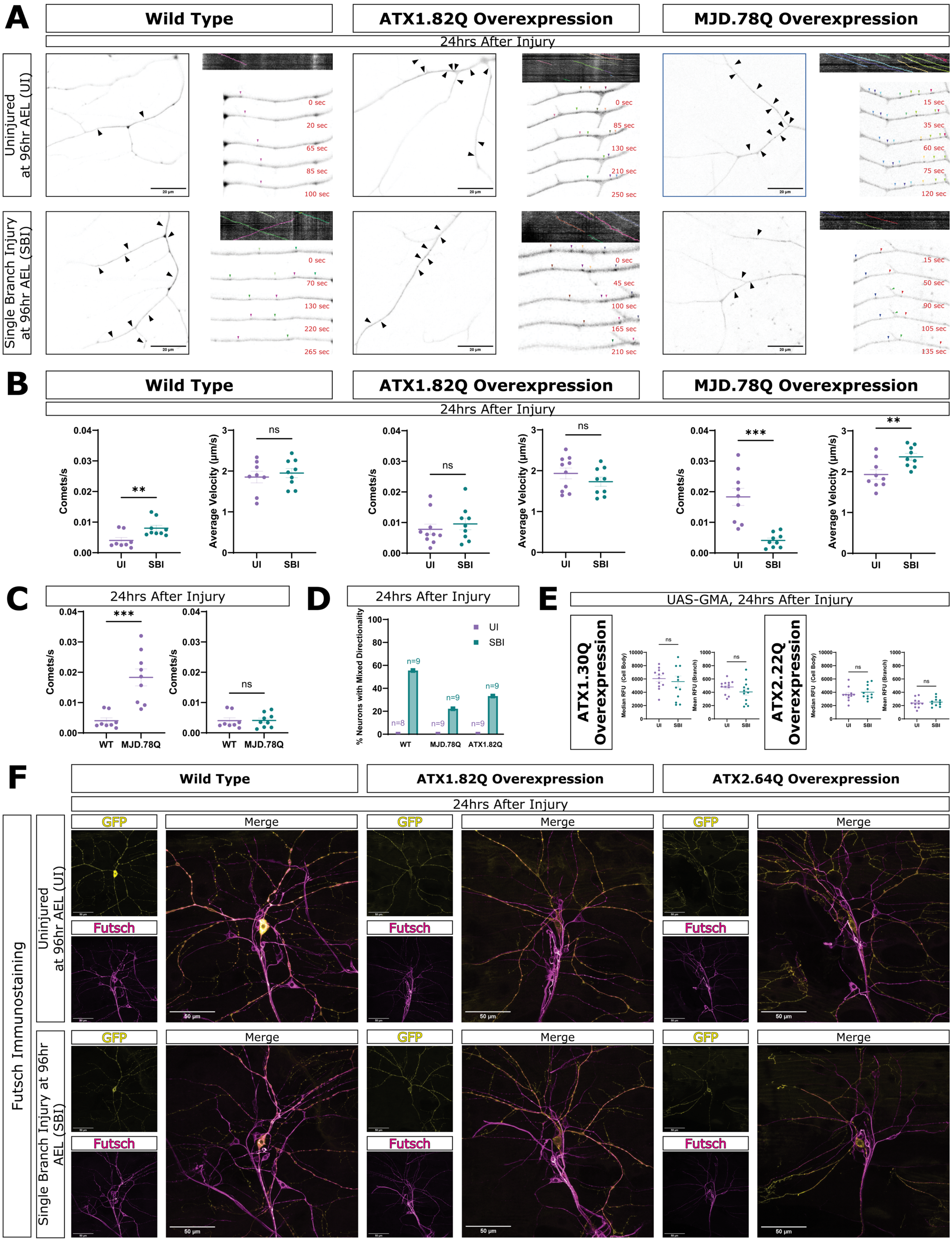

Supplemental Figure 10. Neither microtubule composition nor dynamics are rescued in single branch injured pathogenic polyQ neurons and f-actin is not affected in single branch injured non-pathogenic polyQ neurons. A) Snapshots of EB-1 comet tracking movies for uninjured and single branch injured WT and ATX1.82Q and MJD.78Q overexpression neurons at 24 hrs after injury. Arrows indicate individual EB-1 comets and colored arrows mark the same comet at different time points. Scale bar 20 μm. B) Comets/s for a 10 μm length of dendrite and average comet velocity between UI and SBI neurons for WT and ATX1.82Q and MJD.78Q overexpression neurons at 24 hrs after injury. Welch's t-test. C) Comparison of comets/s for a 10 μm length of dendrite between uninjured WT (purple) and uninjured MJD.78Q neurons (purple) and comparison of comets/s for a 10 μm length of dendrite between uninjured WT (purple) and SBI MJD.78Q overexpression neurons (green) at 24 hrs after injury. Welch's t-test. D) % neurons with mixed EB-1 comet directionality (retrograde, anterograde) at 24 hrs after injury for WT and ATX1.82Q and MJD.78Q overexpression neurons. Values are plotted as percentage of all movies per injury type and genotype. Sample size n represents the total number of neuron movies for each injury type and genotype. E) Median cell body RFU and mean branch RFU between UI and SBI neurons for neurons overexpressing ATX1.30Q and ATX2.22Q. Welch's t-test. F) Immunostaining for GFP (pseudo-color yellow) and futsch (pseudo-color magenta) of uninjured and single branch injured WT and ATX1.82Q and ATX2.64Q overexpression neurons. Scale bar 50 μm.

Supplemental Figure 11. Single primary dendrite branch injury causes neuroprotection in neurons overexpressing pathogenic polyglutamine proteins, while axotomy, terminal branch injury, and near branch injury do not.

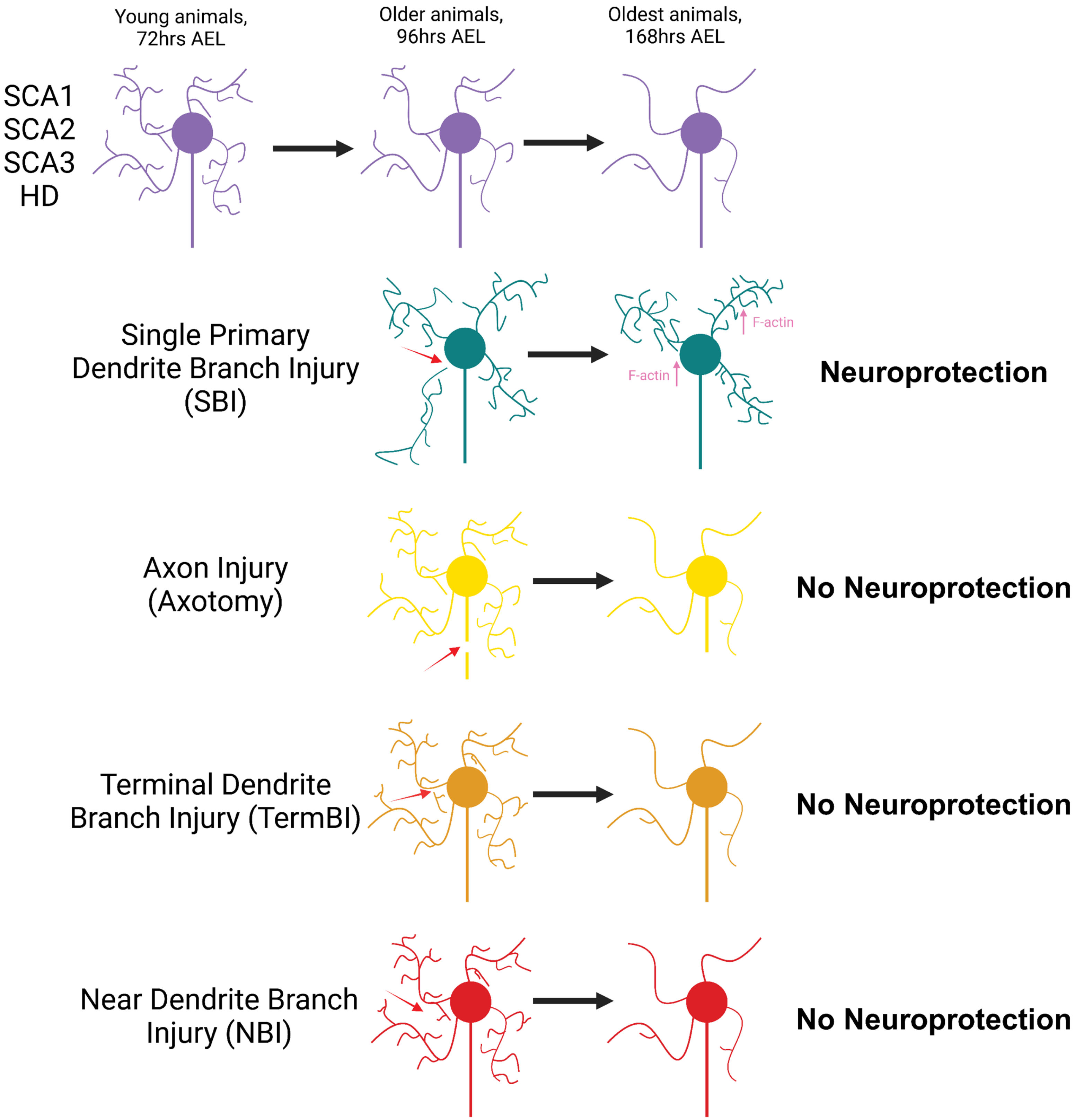

Supplemental Figure 11. Single primary dendrite branch injury causes neuroprotection in neurons overexpressing pathogenic polyglutamine proteins, while axotomy, terminal branch injury, and near branch injury do not. Graphical abstract representing that neurons modelling SCA1, SCA2, SCA3, and HD will degenerate dendrites over time. However, injury to a single primary dendrite branch (SBI) causes neuroprotection of these dendrites which is mechanistically tied to increased f-actin in the dendrite branches and cell body. In contrast, injury to the axon (axotomy), injury to a terminal dendrite branch (TermBI) or injury near a dendrite branch (NBI) do not lead to neuroprotection.

Supplemental methods: Key resources table

REAGENT or RESOURCE	SOURCE	IDENTIFIER
Antibodies		
Mouse anti-futsch	DSHB	RRID:AB_528403
Rabbit anti-GFP	Moecular Probes (Invitrogen), Thermo Fisher Scientific	RRID:AB_221570
Goat anti-Rabbit IgG (H+L) Cross-Adsorbed ReadyProbes Secondary Antibody, Alexa Fluor™ 488	Thermo Fisher Scientific	RRID:AB_2556544
Goat anti-Mouse IgG (H+L) Highly Cross-Adsorbed Secondary Antibody, Alexa Fluor™ Plus 647	Thermo Fisher Scientific	RRID:AB_2633277
**Chemicals, peptides, and recombinant proteins**		
Paraformaldehyde, 4% in PBS	Thermo Fisher Scientific	Cat# J61899-AP
Triton™ X-100 Surfact-Amps™ Detergent Solution	Thermo Fisher Scientific	Cat# 85112
Gibco™ PBS 1X, pH 7.4	Thermo Fisher Scientific	Cat# 10010031
Gibco™ Horse Serum, New Zealand origin	Thermo Fisher Scientific	Cat# 16050122
Glycerol ReagentPlus®, ≥99.0% (GC)	Sigma Aldrich	Cas# 56-81-5
Isoflurane	Piramal, Midwest Vet Supply	Item# 193.33165.3
GeneMate LE Quick Dissolve Agarose	VWR	Cat# E3119-500
**Experimental models: Organisms/strains**		
Drosophila: w[1118]; P{w[+mC]=UAS-Hsap\ATX1.82Q}M6	Fernandez-Funez et al.[44]	RRID:BDSC_33818 FBti0016907
Drosophila: w[*]; P{w[+mC]=UAS-hATXN3.tr-Q78}c211.2	Warrick et al.,Tsou et al. [41,53]	RRID:BDSC_8150 FBti0040564
Drosophila: w[*]; P{w[+mC]=UAS-EB1-GFP}3	Jonathan Scholey[114]	RRID:BDSC_35512 FBti0141213
Drosophila: w[*]; P{w[+mC]=UAS-hMAPT.0N3R.Fc}8	Fabian Fieguin[63]	RRID:BDSC_93609 FBtp0149373
Drosophila: y[1] w[*]; P{w[+mC]=GAL4-ninaE.GMR}12/CyO; P{w[+mC]=UAS-Hsap\ATX1.30Q}F6	Fernandez-Funez et al.[44]	RRID:BDSC_39739 FBti0016905
Drosophila: w[*]; P{w[+mC]=UAS-hATXN3.tr-Q27}N18.3d	Nancy Bonini[55]	RRID:BDSC_8149 FBti0040568
Drosophila: w[1118]; P{w[+mC]=UAS-GMA}3	Dan Kiehart[113]	RRID:BDSC_31776 FBti0131132
Drosophila: UAS-CD4-tdTomato	Han et al. [110]	RRID:FlyBase_FBtp 0068019
Drosophila: UAS-Hsap\HTT231NT.128Q	Juan Botas, Branco et al.[52]	RRID:FlyBase_FBal 0368452
Drosophila: Gal4^2-21	Cheng et al.[112]	RRID:FlyBase_FBal 0328157
Drosophila: Ppk-cd4-tdGFP	Han et al.[110]	RRID:FlyBase_FBti0 143431
Drosophila: ppk-gal4	Grueber et al.[111]	RRID:FlyBase_FBti0 127690
Drosophila: ppk-cd4-tdTomato	Han et al.[110]	RRID:FlyBase_FBtp 0068014

Drosophila: UAS-ATXN2-CAG-64	Nancy Bonini, McGurk et al.[50]	RRID:FlyBase_FBtp0157695; RRID:FlyBase_FBal0386141
Drosophila: UAS-ATXN2.22Q	Kim et al.[54]	RRID:FlyBase_FBti0201755 RRID: BDSC_79594
Drosophila: Gal-4^19-12>cdf-tdGFP	Rumpf et al.[115]	RRID:FlyBase_FBti0148308
Drosophila: WeeP304(tau-GFP)	Melissa Rolls, Stone et al.[71]	RRID:FlyBase_FBti0132768; RRID:FlyBase_FBal0249389
Drosophila: Canton-S	Lab stock	RRID:FlyBase_FBsn0000274
**Software and algorithms**		
ImageJ	Wayne Rasband	RRID:SCR_003070 https://imagej.net/ij/
Adobe InDesign	Adobe	RRID:SCR_021799 https://www.adobe.com/products/indesign.html
Graphpad Prism	Graphpad	RRID:SCR_002798 https://www.graphpad.com/features
Zen Microscopy Software	Zeiss	RRID:SCR_013672
Microscoft Excel	Microsoft	RRID:SCR_016137 https://www.microsoft.com/en-us/microsoft-365/excel
KymoButler	Jakobs et al.[107]	RRID:SCR_021717 https://www.wolframcloud.com/objects/deepmirror/Projects/KymoButler/KymoButlerForm
**Other**		
LSM 700 Fluorescent Confocal Microscope	Zeiss	RRID:SCR_017377
LSM 780 Fluorescent Confocal Laser Scanning Microscope	Zeiss	RRID:SCR_020922
LSM 980 Fluorescent Confocal Microscope with Airyscan 2	Zeiss	RRID:SCR_025048
MAI TAI™ BB 990G 2-photon laser	Spectra-Physics	Model BB 990G
MAI TAI™ EHP 1040S 2-photon laser	Spectra-Physics	Model EHP 1040S
Dissecting Microscopes (Zeiss Stemi 2000, Nikon SMZ-10A, Olympus SZ60	Zeiss, Nikon, Olympus	Models Stemi 2000; SMZ-10A; SZ60
Fiber-Lite Mi-150 Illuminator Series,150w Halogen light source (MI150 Mi 150)	Dolan-Jenner Industries	Cat# 660000391010
Eppendorf™ and Pipetman Micropipettes	Thermofisher Scientific, Sigma Aldrich	Cat# 3123000020; Cat# 3123000098; Cat # 3123000055; Cat# FA10006M

Epredia™ Richard-Allan Scientific™ Cover Glass 22mmx22mm	Thermo Fisher Scientific	Cat# 22-050-235
Fisherbrand™ Premium Plain Glass Microscope Slides	Thermo Fisher Scientific	Cat# 12-544-1
LEICA MZ FLIII Fluorescent sorting microscope	LEICA	Model MZ FLIII
Super High Pressure Mercury Lamp Power Supply	Nikon	Model HB-10101AF
Parafilm® M Sealing Film	Sigma Aldrich	Cat# HS234526B-1EA
Syringe, 10 ml, 5ml	Thermo Fisher Scientific	Cat# 14-955-458; Cat# 14-955-459
Drosophila food	UCI Fly Food Kitchen	N/A
Yeast, Active, Dry	Fisher Science Education	Cat# S25632
Drosophila Incubator	Percival	https://www.percival-scientific.com/product-category/drosophila/
Welch's 100% Grape Juice	Welch's	WEL35400
Fisherbrand™ Round Bottom Disposable Borosilicate Glass Tubes with Plain End	Thermo Fisher Scientific	Cat# 14-961-27
